# Fault Diagnosis of the Rolling Bearing by a Multi-Task Deep Learning Method Based on a Classifier Generative Adversarial Network

**DOI:** 10.3390/s24041290

**Published:** 2024-02-17

**Authors:** Zhunan Shen, Xiangwei Kong, Liu Cheng, Rengen Wang, Yunpeng Zhu

**Affiliations:** 1 School of Mechanical Engineering and Automation, Northeastern University, Shenyang 110819, China; 2 Key Laboratory of Vibration and Control of Aero-Propulsion System, Ministry of Education, Northeastern University, Shenyang 110819, China; 3 Liaoning Province Key Laboratory of Multidisciplinary Design Optimization of Complex Equipment, North-Eastern University, Shenyang 110819, China; 4 Dahua Technology Co., Ltd., Hangzhou 310053, China; 5 School of Engineering and Materials Science, Queen Mary University of London, London E1 4NS, UK

**Keywords:** rolling bearing, intelligent fault diagnosis, adversarial generative network, multi-task learning, semi-supervised learning

## Abstract

Accurate fault diagnosis is essential for the safe operation of rotating machinery. Recently, traditional deep learning-based fault diagnosis have achieved promising results. However, most of these methods focus only on supervised learning and tend to use small convolution kernels non-effectively to extract features that are not controllable and have poor interpretability. To this end, this study proposes an innovative semi-supervised learning method for bearing fault diagnosis. Firstly, multi-scale dilated convolution squeeze-and-excitation residual blocks are designed to exact local and global features. Secondly, a classifier generative adversarial network is employed to achieve multi-task learning. Both unsupervised and supervised learning are performed simultaneously to improve the generalization ability. Finally, supervised learning is applied to fine-tune the final model, which can extract multi-scale features and be further improved by implicit data augmentation. Experiments on two datasets were carried out, and the results verified the superiority of the proposed method.

## 1. Introduction

As a general-purpose component, rolling bearings are widely used in various rotating mechanical equipment. Defects may occur to the bearings during operation, which may ultimately cause damage to the equipment [1,2]. Fault diagnosis of rolling bearings can effectively help prevent safety accidents and economic losses. For instance, the statistic shows that bearing failure accounted for more than 21% of all failures in electrical machines [3]. In the past, the fault diagnosis of rolling bearings was often realized by physical models [4]. However, the oversimplification and low accuracy of physical models make it impossible to apply to the increasingly complex modern industrial system. With continuous improvements in computer processors and sensor technologies (vibration sensor, acoustic sensor, etc.), researchers have summarized the diagnosis scheme into two solutions based on vibration and acoustic. These two solutions can be implemented alone or combined with deep learning methods based on historical data. It provides a new direction for accurate bearing fault diagnosis [5,6] and accelerates the use and development of fault diagnosis tools [7].

The distinct advantages of deep learning over other machine learning methods include its great learning capacity, more powerful feature extracting ability and faster data processing ability [8]. With these advantages, deep learning models, such as convolutional neural networks (CNN) and recurrent neural networks (RNN), have achieved excellent performance in the fields of image processing, natural language processing, etc. [9]. Researchers have also tried to apply deep learning methods to achieve a high-accuracy diagnosis of bearing faults. Eren et al. [10] used one-dimensional CNN and raw vibration signals for bearing fault diagnosis. Liu et al. [11] reported lightweight CNN to perform bearing fault diagnosis under variable operating conditions, and Luo et al. [12] employed a semi-supervised autoencoder (AE) to solve the diagnosis problem when the labeled samples were insufficient.

Although previous research has improved diagnostic accuracy to a certain extent, the information and features of historical data have not been fully excavated and utilized, hindering the further improvement of these methods. In particular, rolling bearing fault diagnosis models based on deep learning are faced with the following challenges. (1) The global features of vibration signals are difficult to extract. Vibration signal data belongs to one-dimensional time series data, up to several thousand dimensions in the time domain. Compared to image data, the structural characteristics of the vibration signal make the receptive field (RF) of CNN smaller. In image processing, when a 5 × 5 convolution kernel is used to stack a 10-layer network, the receptive field of the output layer can reach 1681. However, when the 1 × 5 convolution kernel is stacked with ten layers, the RF can only reach 41 for the vibration signal. Therefore, it is difficult to explore the global features of the vibration signal using a small-size convolution kernel. (2) The large-sized convolution kernel and the deep network structure can enhance the global feature extraction ability but also increase the number of parameters and calculations and thus have a risk of overfitting. Moreover, deep CNNs are laborious to train due to gradient explosion or vanishing. (3) The task of fault diagnosis is unitary. In image processing, diversified learning tasks such as classification, object detection, semantic segmentation, and text annotation can assist each other [13]. Additional learning tasks can be used as regularization items or pre-training methods [14]. In contrast, only one classification task (fault diagnosis) or one regression task (life prediction) is usually performed in the field of diagnosis.

Considering the above challenges, this study aims to develop a fault diagnosis method that can extract global and local features without increasing the learning parameters and the possibility of overfitting. In addition, multi-task learning methods are considered for effective training.

Multi-scale feature learning and multitask learning are not new in the field of image processing. Multi-scale convolution models, such as Inception [15], have more powerful feature extraction and generalization capabilities than single-scale convolution models. Dilated convolution can improve the receptive field of the convolution kernel while keeping the number of parameters consistent [16]. GAN is a deep learning framework proposed by Goodfellow et al. in 2014 [17]. GANs perform unsupervised learning through a binary game to obtain a generative model. Auxiliary classifier generative adversarial networks (ACGANs) are a variant of GANs [18]. ACGAN consists of two components: the discriminator and the generator. The generator randomly samples from random noise and classification labels to generate new fake samples. The discriminator performs source discrimination and classification discrimination on either true or fake samples. Both the discriminator and the generator can effectively improve their performance during the game. Other technologies, such as attention mechanisms and residual structures, are also often used to improve performance. The attention mechanism can improve the representability of CNN and help visualize the learning process [19]. The residual structure effectively resolves the training difficulties of deep networks [20].

Besides the field of image processing, the above-mentioned technologies, such as multi-scale convolution, residual connection, and ACGAN, have also been tried to apply in fault diagnosis, but limitations still exist. Huang et al. [21] employed multi-scale convolution kernels to extract features of bearing faults. However, the study did not involve dilated convolution to reduce the number of parameters and calculations, and a large number of training samples were needed to ensure the accuracy of diagnosis. Li et al. [22] investigated the residual model to accelerate learning but did not utilize multi-scale features or unsupervised learning to improve performance. Shao et al. [23] employed ACGAN for data augmentation while ignoring the discriminator for diagnosis tasks. Huang et al. [24] and Wang et al. [25] adopted an attention mechanism-based model to extract fault features. However, the studies did not reveal the importance of multi-scale features through the attention mechanism.

Considering the current methods’ limitations, this study proposes a new method based on deep learning for bearing fault diagnosis. Different from the previous methods using artificial features as input, a multi-scale dilated convolution kernel is designed in our proposed method to extract features from the raw signal adaptively, and unsupervised learning is used to improve model performance. 

The main contributions of the proposed method can be highlighted as follows:

(1) Multi-scale and multi-dilation rate convolution kernels are used to extract both the global and local features of the raw signals. The receptive field is improved while not increasing the number of parameters significantly or causing serious overfitting.
(2) The channel attention mechanism is employed to illustrate the importance of features at different scales. Features of different scales have different contributions to diagnostic tasks. The channel attention mechanism adaptively learns the importance of various features, and the role of features at various levels and scales could be revealed. Therefore, the proposed method enhances the interpretability of the fault diagnosis model.
(3) A multi-task learning model suitable for bearing fault diagnosis is established. The new method uses an unsupervised learning task to strengthen the feature extraction capabilities of the diagnostic model. Essentially, the proposed method has implicit data augmentation.

The remaining parts of this paper are arranged as follows. Section 2 provides the preliminary details of this study. In Section 3, the proposed method and model are demonstrated in detail. In Section 4, the experimental arrangement and data processing work are described. In Section 5, experimental results on two datasets are provided to illustrate the effectiveness of the proposed method. Finally, the conclusion is drawn in Section 6.

## 2. Related Work

### 2.1. Dilated Convolution

The most obvious characteristics of CNN are weight sharing and local connection. The convolution operation or cross-correlation operation performed by the convolution kernel is a special form of weight sharing and local connection. The most important advantage of weight sharing and the local connection is to reduce the number of parameters. It is easy to get a dilated convolution from the basic convolution. A dilated convolution is suitable for one-dimensional long sequence data, such as vibration signals with a larger receptive field.

**Figure 1:**
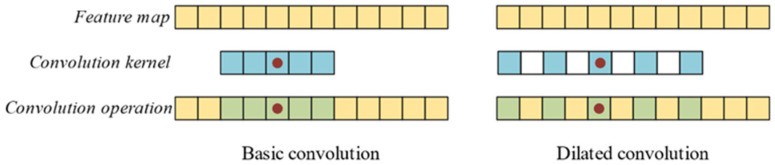
Basic convolution and dilated convolution.

Figure 1 shows a basic convolution kernel with a size of 1 × 5, a dilated convolution kernel with a size of 1 × 5, and a dilation rate of 2. The single-step convolution ranges of the basic convolution and dilated convolution are 5 and 9, respectively. The dilated convolution forces the calculation result of the hole area to be zero, which is an infinitely strong priori assumption like the pooling operation. Therefore, the rationality of the dilated convolution should be verified through practice. When maintaining the same convolution range, the dilated convolution kernel has fewer parameters than the basic convolution kernel. The greater the dilation rate, the fewer the number of parameters. Due to information loss, the hole rate is not always greater or better. For example, when the dilation rate is greater than 1, and the step size is also greater than 1, it may cause some parts of the data to be missed in the calculation, which results in absolute information loss.

### 2.2. Channel Attention Mechanism

**Figure 2:**
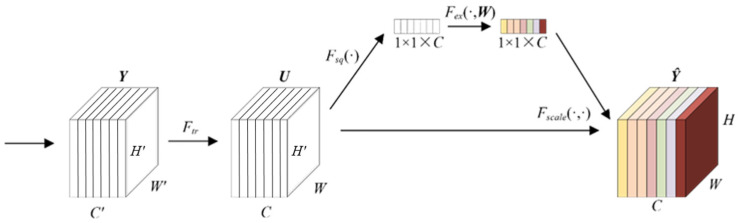
Structure of Squeeze-and-Excitation.

During image processing, the features of different channels are of different importance for the task. Similarly, the features of different time scales have different significance for vibration signals. A Squeeze-and-Excitation (SE) [26] structure is proposed to learn the degree of correlation among channels. SE is a simple yet effective feedforward CNN attention module, and it can recalibrate the feature map extracted by the convolution kernel to improve CNN’s feature extraction and representation capabilities [27]. As shown in Figure 2, SE uses a global pooling operation to compress the channel information of feature ***U***. The fully connected layer or convolutional layer is used to learn the importance of each channel adaptively. The activation value output by the activation function recalibrates the feature ***U***. In (1), ***Y*** and *F_tr_*(·) are the features and transformations before the SE structure, respectively. ***U*** is the input of the SE structure. In (2), *F_sq_*(·) is the global pooling operation. *F_ex_*(·, ***W***) represents the fully connected layer or convolutional layer. ***W*** is the parameter of the fully connected layer or convolutional layer. *F_scale_*(·,·) indicates the channel calibration for ***U***. The original SE uses global average pooling (GAP) to compress the features of each channel. Another commonly used global pooling is global maximum pooling (GMP).

(1) $$U=F_{tr}(Y)$$

(2) $$\hat{Y}=F_{\mathrm{scale}}(U,F_{\mathrm{ex}}(F_{\mathrm{sq}}(U),W))$$

### 2.3. Residual Structure

**Figure 3:**
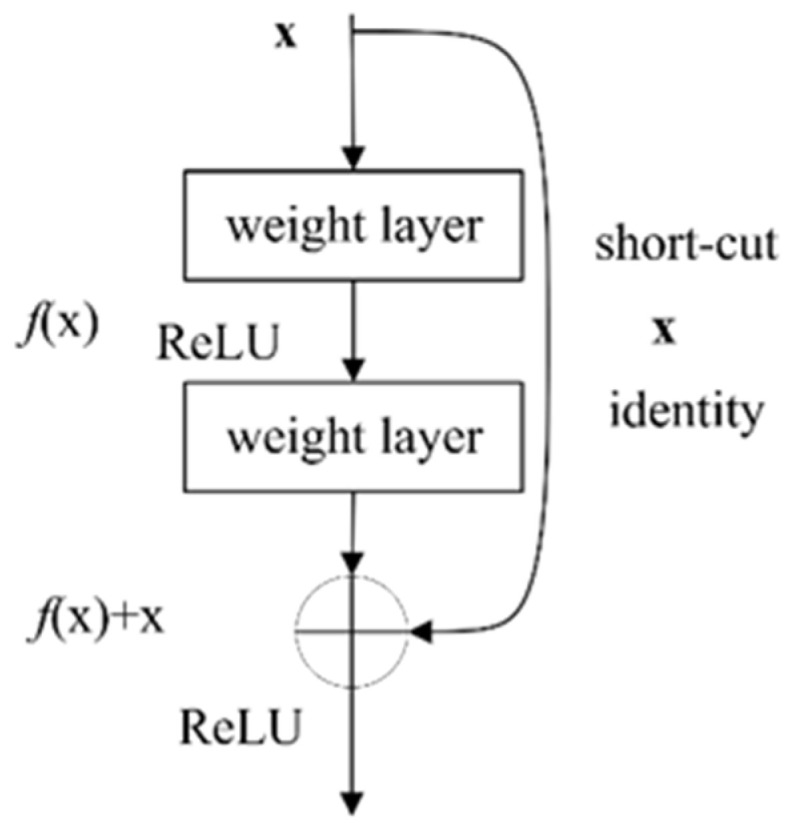
Structure of original residual.

Deep and narrow networks reduce the number of parameters and model scales and tend to have better generalization capabilities than shallow and wide networks. However, the phenomenon of gradient explosion or vanishing makes deep networks more difficult to train. ResNet proposes a residual structure based on identity transformation (as shown in Figure 3), which effectively alleviates the phenomenon. The core of the residual module is to use a *short-cut* connection [28]. From the perspective of backpropagation, the *short-cut* connections shorten the chain derivation path and alleviate the gradient explosion or vanishing.

### 2.4. ACGAN

ACGAN is a semi-supervised generative adversarial network that adds an auxiliary classifier based on the original GAN. It improves the stability of the training process and the quality of the generated samples. The generator *G* in ACGAN takes random labels and noise as input and generates fake samples to confuse discriminator *D*. *D* distinguishes the source and classification of the samples. Distinguishing the source is called the main task of ACGAN, and distinguishing the classification is called the auxiliary classification task. Denote the losses of the two tasks as *Ls* and *Lc*, respectively. They can be calculated as:

(3) $$L_{S}{=E}_{x\sim p_{data}(x)}[\log P(S=real|x)]{+E}_{z\sim p_{z}(z)}[\log(P(S=fake|x=G(z)))]$$

and

(4) $$L_{C}{=E}_{x\sim p_{data}(x)}[\log P(C=c|x)]{+E}_{z\sim p_{z}(z)}[\log P(C=z|x=G(z))]$$

In (3) and (4), $p_{data}(x)$ is the probability distribution of the real sample *x*. $p_{z}(z)$ is the prior probability distribution of label *z*. *G*(·) represents the transformation function of *G*. *c* represents the label of *x*. *S* and *C* represent the prediction of sample source and classification, respectively. S∈{real, fake}, $C\in \{c_{0},c_{1},\ldots \}$. In the training process, *D* is trained by maximizing *L_s_ + L_c_*, and *G* is trained by maximizing *L_s_ − L_c_*. Compared with GAN, ACGAN has the following advantages: (1) The learning stability is significantly enhanced; (2) The quality of generated samples is significantly improved; and (3) Both the sample-generating ability of *G* and the feature-extracting ability of *D* are improved.

## 3. Proposed Method

Firstly, this study constructs a convolution module based on the dilated convolution, SE, and residual structure to extract features on various time scales. Then, a classifier generative adversarial network (CGAN) based on the idea of ACGAN for fault diagnosis is designed. In CGAN, *G* and *D* use the constructed convolution module or its internal sub-modules. Finally, two stages of learning are employed to improve the diagnostic accuracy of the proposed model. The first stage is multi-task learning based on CGAN. The second stage is fine-tuning based on supervised learning.

### 3.1. Multi-Scale Dilated Convolution SE Residual Module

**Figure 4:**
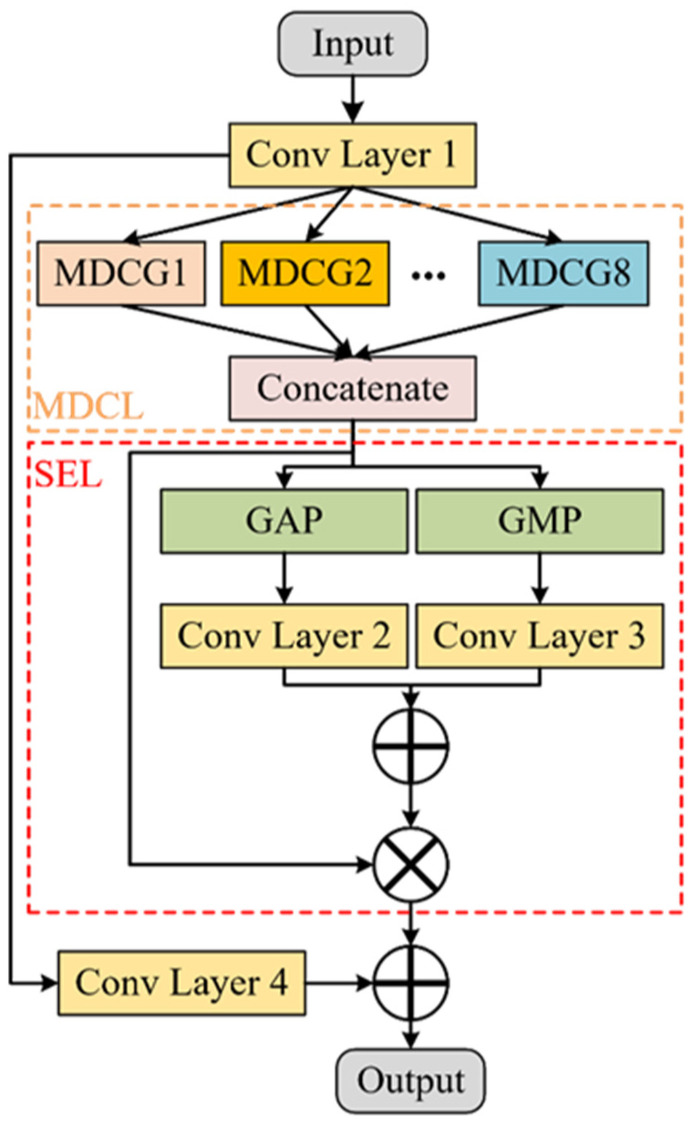
Structure of MDC-SE-ResBlock. MDCL and SEL represent the MDC layer and SE layer, respectively.

As shown in Figure 4, the proposed convolution module embeds the multi-scale dilated convolution (MDC) layer and SE module into the residual module. Therefore, it is called a multi-scale dilated convolution squeeze-and-excitation residual block (MDC-SE-ResBlock). The MDC layer comprises eight groups of dilated convolution kernels with different scales and dilation rates, called multi-scale dilated convolution group (MDCG). The detailed parameters are shown in Table 1. Compared with the original SE module, the SE module in this paper uses both GAP and GMP to form a global pooling layer. In MDC-SE-ResBlock, Conv-Layer1 and Conv-Layer4 are added to maintain the consistency of the feature size and the number of channels.

As shown in Table 1, the convolution range of MDCGs ranges from 3 to 111. Since there is a certain degree of information loss in dilated convolution [29], this new model sets the step size of all MDCGs to 1 to reduce information loss. The step size of Conv Layer 1 and Conv Layer 4 is set to 2 to achieve down-sampling and information compression. In MDC-SE-ResBlock, the Leaky Rectified Linear Unit (LeakyReLU) [30] is selected as the activation function. Batch Normalization (BN) [31] and Dropout [32] are also used to stabilize the learning process and strengthen regularization, respectively.

The MDC, SE, and residual modules constructed by MDC-SE-ResBlock all have distinct advantages. Dilated convolution can reduce network parameters and prevent serious overfitting while maintaining a larger RF. Therefore, the dilated convolution is particularly suitable for one-dimensional time-series signals. For example, in MDCG8, the basic convolution kernel with the same convolution range contains 111 parameters. MDCG8 contains only 23 parameters, which reduces the number of parameters by 79.28%. The SE module can adaptively learn the importance of features at different scales. The activation values of SE can also be used for visualization. The residual structure introduced by MDC-SE-ResBlock can alleviate gradient explosion or vanishing effectively.

### 3.2. Classifier Generative Adversarial Net (CGAN)

**Figure 5:**
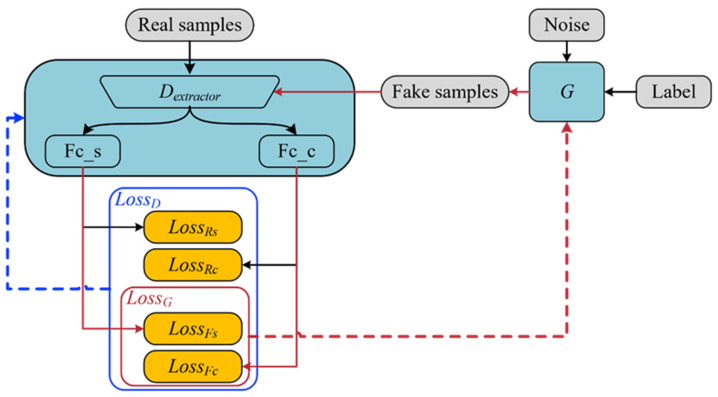
The framework of CGAN.

The goal of ACGAN is to obtain a high-quality *G*, but in this study, we want to learn a discriminator *D*, which will be used as a classification network in the later tasks. The classification task should be the main task, while the source identification task should be the auxiliary task. In this case, we call ACGAN CGAN, and the goal of CGAN is to obtain a stable and reliable fault classification model D. The specific differences between CGAN and ACGAN can be shown in Table 2. Figure 5 illustrates the framework of CGAN. The classification task is achieved by maximizing $Loss_{D}=Loss_{R_{S}}+Loss_{R_{C}}+Loss_{F_{S}}+Loss_{F_{C}}$, while the source identification task is achieved by maximizing $Loss_{G}=Loss_{F_{S}}+Loss_{F_{C}}$.

(5) $$Loss_{R_{S}}=E_{x\sim p_{data}(x)}[\log P(S=real\left|x\right.)]$$

(6) $$Loss_{R_{C}}=E_{x\sim p_{data}(x)}[\log P(C=c\left|x\right.)]$$

(7) $$Loss_{F_{S}}=E_{z\sim p_{z}(z)}[\log(P(S=fake\left|x=G(z)\right.))]$$

(8) $$Loss_{F_{C}}=E_{z\sim p_{z}(z)}[\log P(C=z\left|x=G(z)\right.)]$$

**Figure 6:**
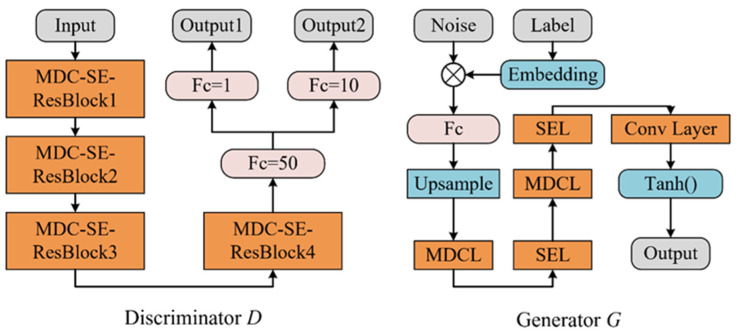
The structures of *D* and *G*.

In CGAN, the discriminator *D* and generator *G* structures are shown in Figure 6. *D* is cascaded with 4 MDC-SE-ResBlocks. The output layer of *D* has an RF of about 500 to the input layer, which can effectively extract global features. MDC-SE-ResBlock is not used directly in *G*, and two sets of MDC-SE structures in series are used instead. There are two reasons for the design of *G* in this paper. On the one hand, the learning effect of *G* mainly depends on *D*, so the residual structure is not necessary for *G*. On the other hand, after many attempts in this study, it is found that the effect of directly using MDC-SE-ResBlock is not as good as the current structure. Among the proposed *D* and *G*, LeakyReLU, Dropout, and Batch Normalization are still used.

### 3.3. Learning Strategy

This paper proposes a two-stage learning strategy to train the fault diagnosis model. Only labeled samples are used in the entire learning process. In Stage 1, the CGAN learning strategy is used for semi-supervised learning. In Stage 2, *D* again uses the labeled samples for supervised fine-tuning.

#### 3.3.1. The First Stage of Learning

The learning strategy of CGAN is used to pre-train the diagnostic model *D* so as to better initialize the parameters. For supervised learning tasks, direct learning supervised tasks can easily fall into a poor local optimum, which leads to poor generalization ability. An appropriate pre-training method can constrain the parameters to the vicinity of the global optimum point. The CGAN learning strategy has the following advantages: (1) After unsupervised learning, *D* obtains a good representation of the raw data; (2) When the Nash equilibrium is reached, it is equivalent to carrying out implicit data augmentation that the *D* classifies the samples generated by *G*; and (3) Supervised learning carried out by *D* can overcome the blindness of unsupervised learning. The first stage of learning is both multi-task learning and semi-supervised learning. The learning process of the first stage is cyclically updating the parameters in *D* and *G*. The specific learning process is shown in Algorithm 1. In this paper, the Adam optimizer is used for gradient descent. Stochastic gradient descent (SGD) or RMSProp algorithm can also be used as an alternative.
**Algorithm 1** Training process of the first learning stage**Initialization:** *lr*, *delta*, *n*, *EPOCH*, *ITERATION*. *lr* is the initial learning rate. *delta* is the corresponding decay rate. *n* is the mini-batch size, and *EPOCH* is the total number of times the data set will be traversed. *ITERATION* is the number of training iterations for one epoch.1:**For** *i* in range(0, *EPOCH*) **do:**2:    **For** *j* in range(0, *ITERATION*) **do:**3:            Sample $\left\{(z^{\left(1\right)},y_{fake}^{\left(1\right)}),\ldots ,(z^{\left(n\right)},y_{fake}^{\left(n\right)})\right\}$ from $p_{z}(z)$ and $p_{y}(y)$.4:            Generate $\left\{(x_{fake}^{\left(1\right)},y_{fake}^{\left(1\right)}),\ldots ,(x_{fake}^{\left(n\right)},y_{fake}^{\left(n\right)})\right\}$ by ${\left\{G(z^{\left(k\right)},y_{fake}^{\left(k\right)})\right\}}_{k=1}^{n}$.5:            Sample $\left\{(x_{real}^{\left(1\right)},y_{real}^{\left(1\right)}),\ldots ,(x_{real}^{\left(n\right)},y_{real}^{\left(n\right)})\right\}$ from $p_{data}(x,y)$.6:         Optimizing $Loss_{D}$ by updating the *D.*7:         Optimizing $Loss_{G}$ by updating the *G*.8:     
**end for**
9:     
lr←lr×delta.
10:**end for**

#### 3.3.2. The Second Stage of Learning

To further improve the performance in supervised tasks, supervised fine-tuning is adopted to train *D* in Stage 2. Theoretically, one or more from the multiple trained *D* that finally reach the Nash equilibrium can be selected, and then the selected model is trained again using the training data. In this paper, we simply choose a *D* with a smaller loss in the equilibrium stage according to the loss curves of *D* and *G*. A lower learning rate is employed in this stage to avoid wasting the previous learning. Only labeled samples are used for learning, which is different from many semi-supervised methods that require many unlabeled samples [33].

## 4. Experimental Setup

### 4.1. Experiment on the CWRU Dataset

The open dataset provided by Case Western Reserve University (CWRU) [34] is referred to for the experimental validation. Methods in the literature [24] are also tested on the CWRU dataset. In this study, the faults are divided according to the size and location of the defects. Data under different operating conditions but belonging to the same fault are combined to ensure there are enough samples for various health states. All the data are divided into a training set for model training and a test set for model testing. There are 10 health conditions in total to form a 10-class classification task. Table 3 gives detailed information about this dataset. In Table 3, N represents the normal health condition. IR1~IR3, BF1~BF3, and OR1~OR3 represent the inner ring raceway defect, the ball defect, and the outer ring raceway defect with different degrees of damage, respectively.

### 4.2. Experiment on the Self-Built Dataset

**Figure 7:**
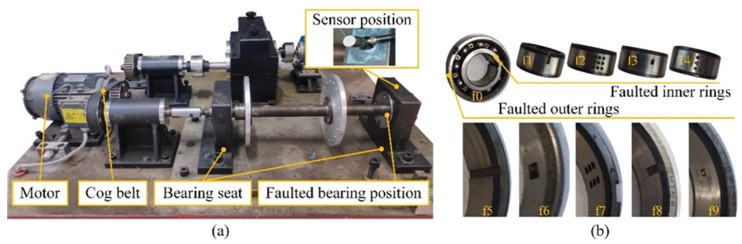
Information on the self-built dataset. The test rig (**a**) and faulted bearings (**b**). f0~f9 correspond to fault0~fault9. f0 is the bearing under normal health conditions. f1~f4 correspond to four inner rings with different defects. f5~f9 correspond to five outer rings with different defects.

A bearing fault test rig is also designed to validate the effectiveness of the proposed model. The test rig is shown in Figure 7a, which consists of a three-phase synchronous motor, a transmission belt, a double wheel, experimental bearings, etc. During the experiment, the vibration signal was collected under 10 healthy conditions (fault0~fault9) of the cylindrical roller bearing, including four types of inner ring faults, five types of outer ring faults, and one normal state (Figure 7b). All the defects in the bearing are fabricated by electrical discharge machining and are different in size, location, or shape. Speed and load remain consistent to avoid interference from irrelevant factors. The signals of all health conditions are continuously collected for 64 s at a sampling frequency of 19,200 Hz. Each signal is divided into 1200 samples, each 1024 in length. Samples are selected from the dataset to form a training set for model training and a test set for model testing. A series of 10-class classification tasks that vary in the size of the training set are designed to investigate the influence of the number of training samples on the diagnostic performance, as shown in Table 4.

## 5. Results and Analysis

### 5.1. Classification Performance on the CWRU Dataset

**Figure 8:**
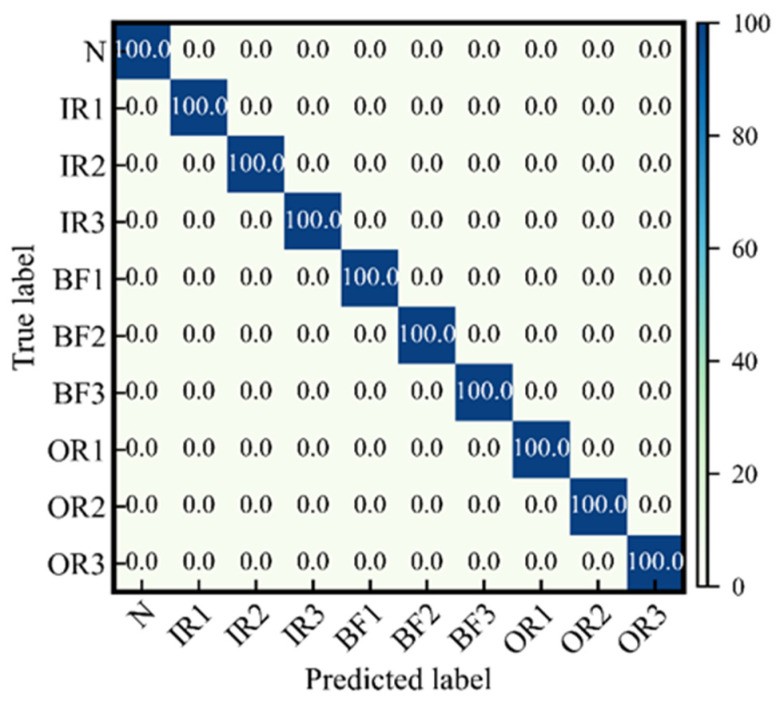
The confusion matrix on the CWRU test set with the proposed method.

The superiority in fault classification of the proposed method is first evaluated on the CWRU open dataset. We conducted a comparative experiment using five popular methods with our own proposed methodology. ICDSVM [35] decomposes vibration signals into intrinsic modal functions through ensemble empirical modal decomposition, extracts their multi-scale intrinsic features, and then uses support vector machines optimized for inter-cluster distance to identify fault types. MC-CNN [21] is a multi-scale cascaded CNN that avoids the local optimization problem of CNN and has good performance in bearing fault diagnosis; the multi-scale information was obtained by filters with different scales to input the CNN. FMCNN [36] combines the sparse representation with the feedback mechanism of CNN for fault diagnosis, and in this process, the Wavelet Packet transform is used as the basis function to construct a dictionary with structural effects, and the mixed penalty term is introduced to further optimize the performance of structural sparse representation. The unsupervised learning [37] method is a two-layer neural network for intelligent fault diagnosis utilizes sparse filtering for feature learning of vibration signals and Softmax regression for classifying health conditions; MA-MSCNN [24] innovatively combines multi-scale convolutional layers with a multi-attention mechanism in order to optimize the model’s use of multi-scale information while maintaining both global and local features, and to better utilize the label information for classification, which has achieved advanced good performance in bearing fault diagnosis. The results of the comparative experiments are listed in Table 5. The confusion matrix of the predicted results of the proposed method is shown in Figure 8. The proposed method achieves the best performance with the highest diagnostic accuracy of 100.0%. It is important to know that the high recognition accuracy of CNNs is based on a complex deep network structure, and therefore a large number of training samples are needed to improve the generalization ability of the model; however, in practice, the data applied to machinery fault diagnosis is limited, which is not entirely realistic. Our proposed method of combining CGAN has far fewer training samples than these comparative methods, and the accuracy is far superior. We noticed that the methods in the references either use explicit data augmentation methods to expand the data set or use many unlabeled samples. In contrast, the proposed method has obvious advantages.

### 5.2. In-Depth Analysis of the Proposed Method Based on the Self-Built Dataset

The data in the CWRU dataset are relatively clean with weak noise, thus the signals in this dataset are very consistent, and it is easy to achieve good performance in the fault diagnosis tasks. Therefore, further experiments on the self-built dataset are carried out to make an in-depth analysis of the proposed method.

#### 5.2.1. Two-Stage Learning Process

**Figure 9:**
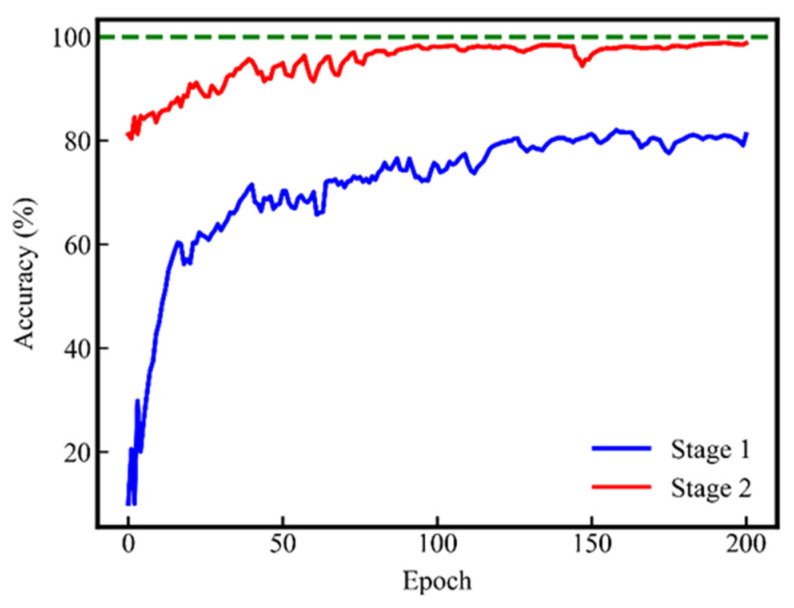
Accuracy curve of the proposed method from task S3.

Task S3 is used to train CGAN. The accuracy curve is shown in Figure 9. In Stage 1, the diagnostic accuracy of *D* gradually increases from 10% to about 80%. The semi-supervised tasks require *D* to learn features to be able to distinguish the real samples from the fake samples. However, these features may be redundant or interfere with the diagnosis task, limiting the accuracy at the first stage. Overall, the semi-supervised task promotes *D* to learn more comprehensive features and makes the parameter value of *D* close to the global optimum of the supervised task.

**Figure 10:**
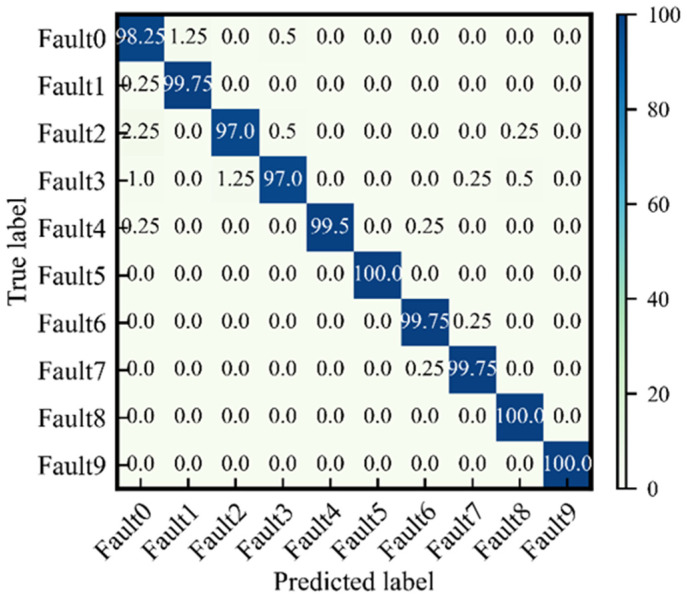
Confusion matrix of the proposed method from task S3.

At the beginning of Stage 2, there is some jitter in the diagnostic accuracy. However, when the epoch is greater than 25, the diagnostic accuracy rate remains above 90%. After stabilization, the accuracy of *D* stays at around 98.5%. The highest accuracy of *D* is 99.1%. The confusion matrix of the highest accuracy model is shown in Figure 10. As mentioned earlier, fault0~fault3 are inner ring faults, and fault5~fault9 are outer ring faults. Figure 10 agrees well with the actual situation. The inner ring faults are more likely to be misclassified with each other. For example, fault0 is misclassified as fault1 or fault2, and fault3 is misclassified as fault0. Similarly, outer ring faults are more likely to be misclassified with others. For example, fault6 is misclassified as fault7, and fault7 is misrecognized as fault6.

#### 5.2.2. Attention Mechanism in MDC-SE-ResBlock

**Figure 11:**
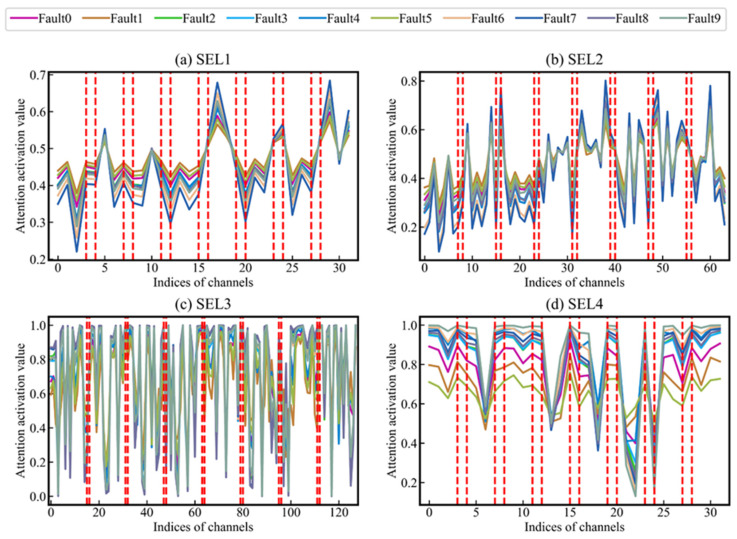
AAV curves of the input fault samples for ten classes in SE layers 1~4.

Figure 11 shows the attention activation value curves of four MDC-SE-ResBlocks used in the proposed method. The attention activation values of the eight groups of MDCG are separated by two red, vertical dashed lines. The importance of each channel feature can be explained by the output value of the activation function (attention activation value, AAV). The value of AAV equal to 1.0 indicates that the SE has performed an identity transformation on the features of the channel and retains all the information of the corresponding channels. The value of AAV is equal to 0.0, which means discarding all the information of corresponding channels. The AAV curves in Figure 11a,b are far away from 1.0 or 0.0, and there are more intersections (circled by the red ellipse), which indicate that SE has played a role in recalibration. A large number of AAV values close to 1.0 and 0.0 in SEL3. Therefore, SEL3 retains the features of some channels completely while discarding the features of others. This shows that there are too many channels in MDCG3, and the number of channels in MDCG3 can be reduced appropriately without degrading the performance. The AAV curve of MDC-SE-ResBlock4 (Figure 11d) has fewer intersections. The AAV curve of each fault shows sufficient differentiation, and the features of various faults are scaled to different intervals. Figure 11d shows that various faults can be distinguished by the features of different channels—the SE module only needs to maintain this differentiation. Figure 11c,d show that the deep SE module is not necessary.

In Figure 11a, the AAV curves of various faults have a large degree of coincidence. This shows that the earlier features are the general features shared by different classes. In the AAV curve of SEL4 (Figure 11d), the overlap of the attention activation value curves of different fault classes is significantly reduced, and there is almost no intersection between the AAV curves. This indicates that the deep features become class-specific. The above conclusions are consistent with previous research conclusions [38,39]. Figure 11a also shows that features of the same scale have different importance for different fault classes. Large-scale features (global features) are even more important for certain classes of faults than small-scale features (local features). For example, in the AAV curve of SEL1, the features of fault7 extracted by MDCG5 and MDCG8 are often retained, while the features extracted by other MDCGs are often discarded.

#### 5.2.3. The effect of Global Features

Another experiment is designed to verify the effect of global features on the accuracy of diagnosis. Model A and Model B in Table 6 are designed to have almost the same framework as *D* in the proposed model. The MDC-SE-ResBlock in Model A only uses MDCG2, while the MDC-SE-ResBlock in Model B uses both MDCG2 and MDCG5. Task S3 is used for five repeated supervised trainings of Model A and Model B. The average accuracy of Model A is 64.86%, and the average accuracy of Model B is 78.44%. The results in Table 6 confirm that global features are very important.

#### 5.2.4. Ablation Experiment

Based on the principle of the controlled variable method, this study performs an ablation experiment to verify the effect of MDC, SE, residual structure, and training method on the final diagnostic accuracy. Models with one sub-structure or training strategy canceled are listed in Table 7. Only MDCG2 is used in the MDC layer of Model1 to verify the effect of multi-scale convolution. The SE layer in MDC-SE-ResBlock of Model2 is canceled to verify the influence of channel attention. In the MDC-SE-ResBlock of Model3, *short-cut* and Conv Layer4 are deleted to verify the function of the residual structure. Model4 uses supervised learning methods for training to verify the effectiveness of the two-stage training strategy. Model5 uses the original GAN for the first stage of training to verify the effectiveness of the training strategy based on CGAN.

**Figure 12:**
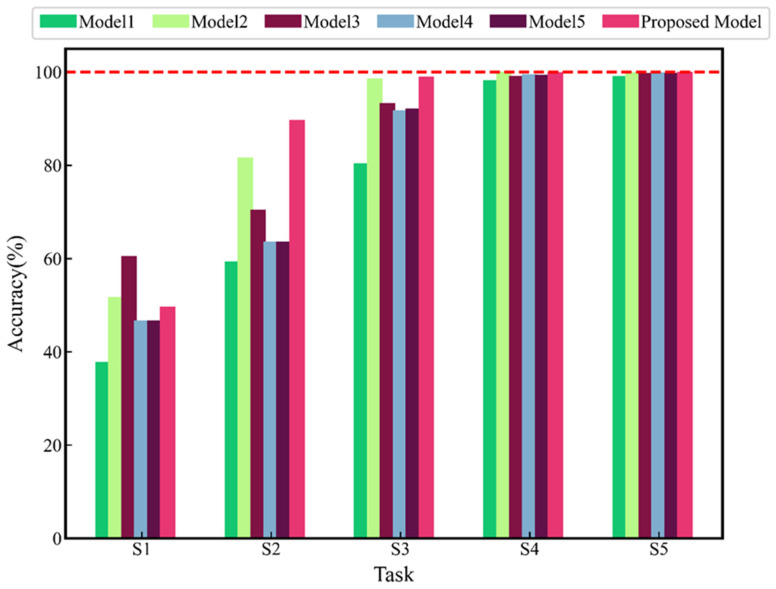
Results of ablation experiment.

The models in Table 7 are used to compare with the proposed method to verify the advantages of the latter in terms of data requirements and generalization capabilities. Tasks S1–S5 are used for five pieces of training of the compared models. The best results of each training session are recorded. The average of the 10 best results is taken as the performance evaluation metrics of each model. The results of diagnostic accuracy are shown in Figure 12, which reflects the superiority of the proposed method. The results illustrate a common phenomenon: as the number of training samples decreases, the accuracy of the diagnostic model decreases to varying degrees. The proposed models all show the best diagnostic effect when trained with Task S2–S5. In addition, in Task S1, Model 2 and Model3 perform the best. When the number of data decreases, Model2 and Model3 perform better because they have fewer parameters with a slighter overfitting problem. In all cases, the training strategy of the proposed method showed the best effect.

In Figure 12, the average accuracy of the proposed method and Model1~Model5 under the five training sets are 87.66%, 75.01%, 86.53%, 84.67%, 80.32%, and 80.34%, respectively. The comparison between the proposed method and Model1 shows the superiority of multi-scale dilated convolution. When the data is insufficient, large-scale dilated convolution can extract significant global features, improving diagnosis accuracy. The comparison between the proposed method and Model2 and Model3 shows that SE and residual structure can improve diagnosis performance. However, when the size of the training set is small (task S1), a serious over-fitting phenomenon is caused by the SE and the residual structure, which overshadows the original advantages. The results of the proposed method and Model4 reflect the superiority of the CGAN training strategy. The final accuracy in task S1 and task S2 for Model5 directly uses the results of Model4 because model collapse occurred in the training of GAN. The comparison between the proposed method and Model5 shows that CGAN is more stable.

**Figure 13:**
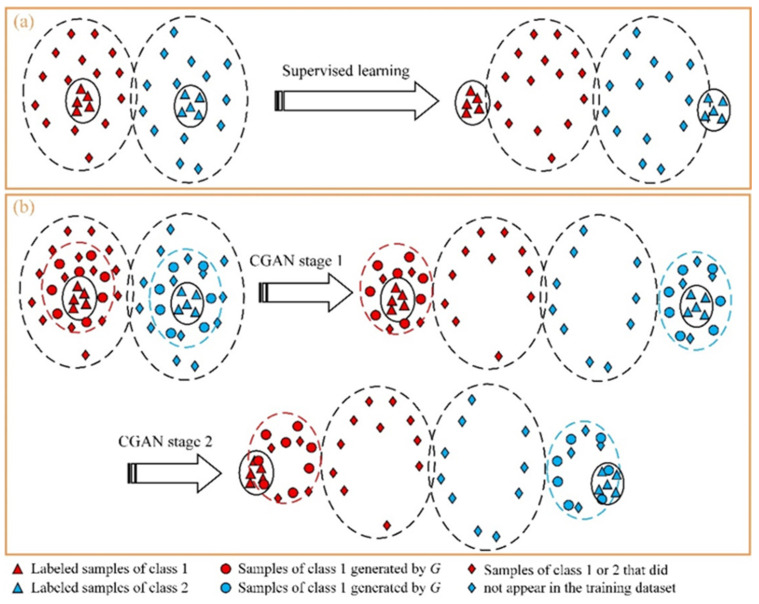
Schematic diagram of the generalization ability improvement of the proposed method. (**a**) The classification process of supervised learning. (**b**) The classification process of proposed methods.

When the first stage of learning reaches the Nash equilibrium, *D* learns the classification task of the samples generated by *G*. In fact, this is implicit data augmentation. Figure 13 shows this process. In the literature [40], this kind of data augmentation is explicitly performed separately. At Stage 1, *D* not only classifies the real samples in the training dataset but also classifies the generated samples by *G*. As shown in Figure 13b, the samples generated by *G* expand the classification scope of *D* so that more true samples that disappear in the training set are correctly classified. Stage 2 further ensures the complete separation of the training set samples. When the number of samples is large enough, *D* learned in Stage 1 has sufficient generalization ability. The accuracy of the first stage of learning of the proposed method in task S5 reaches 99.5%. Note that Stage 2 is not redundant. The a priori learning conveyed in Stage 2 is that the real samples have greater weight than the generated samples for the classification task.

Moreover, when there is a deviation in the learning of Stage 1, Stage 2 can rescue it. On average, the learning in Stage 2 ensures that the final diagnosis effect is at least not worse than the effect when only supervised learning is used. Learning with only supervised learning does not have the same advantages.

#### 5.2.5. Feature Visualization

**Figure 14:**
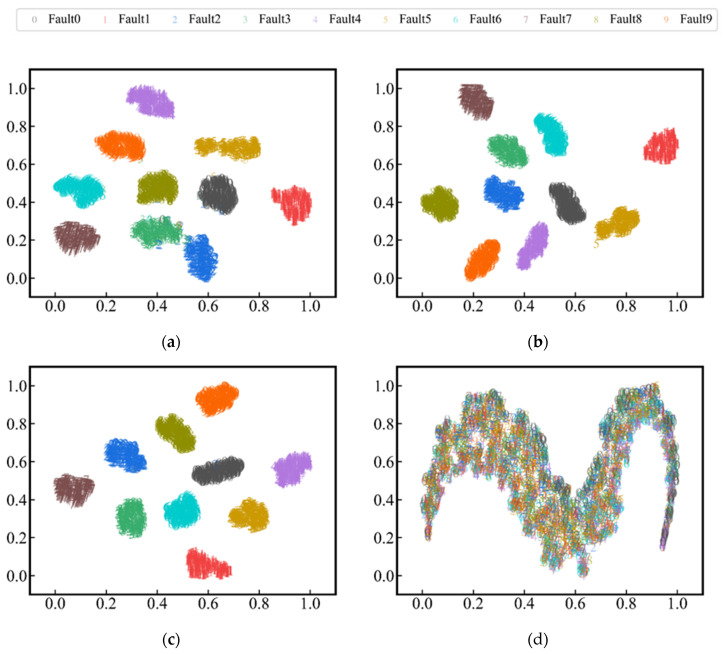
2-D t-SNE visualizations of high-dimensional features (**a**) The proposed method after Stage 1; (**b**) The proposed method after Stage 2; (**c**) Model4; (**d**) Model5 after stage1.

This section uses the t-SNE [41] technique to visualize the features extracted by *D*. The training data and test data from task S3 are used for model training feature visualization, respectively. Figure 14a is the visualization when the first stage of learning with the proposed method is completed. It can be found that various faults have been separated. Figure 14b represents the visualization of different faults when the second stage of learning the proposed method is completed. It can be found that various faults have also been completely separated. Figure 14c shows the visualization of Model4 when the first stage of learning is completed. The intra-class distance is larger than the proposed method, while the inter-class distance is less than the proposed method. Figure 14d shows the visualization of Model5 when the first stage of learning is completed. It can be found that there is no separation of different faults.

Figure 14a,b show that the two stages of learning have different effects. At Stage 1, due to the implicit data augmentation, *D* will expand the sample space of different classes. At Stage 2, *D* further increases the inter-class distance and reduces the intra-class distance of training samples. This further confirms the process shown in Figure 13. Comparing Figure 14a,c, it can be identified that the effect of only supervised learning being used is worse than that of the proposed method. Figure 14d shows that GAN compresses all real samples into a low-dimensional manifold (2-dimensional “M”-shaped surface), so the pre-training method based on GAN can improve the feature-extracting ability of *D* to a certain extent. However, the *D* trained by GAN has not learned the differentiated features of samples belonging to different classes. In addition, *D* only learned the common features of all classes. This can be seen in Figure 14d, where features of different classes are gathered in different manifold areas, and no separation exists at the class-level. Figure 14d also shows that the fault diagnosis method based on manifold learning [42] has practical value. Figure 14a,d jointly verify that CGAN-based learning strategy is superior to GAN.

## 6. Conclusions

This study proposes a novel deep-learning model and training method for bearing fault diagnosis. On the premise of using only label samples, the proposed model and training method can further improve bearing fault diagnosis accuracy. In this study, the comparison with GAN and supervised learning methods validates the advantages of the proposed method in terms of diagnostic accuracy and the size of demanded labeled samples. Moreover, the new model can effectively resolve the challenges of extracting global features, poor learning interpretability, and single learning tasks. Although the proposed method can improve the diagnostic accuracy to a certain level, the generalization ability of this method still needs to be improved in the environment of low signal-to-noise ratio and variable working conditions. Future efforts will be dedicated to improving the accuracy of fault diagnosis under various operating conditions and low signal-to-noise ratio environments.

## Figures and Tables

**Table 1:** Parameter settings of each layer in MDC-SE-ResBlock.

Module	Kernel Size	Padding Size	Dilation Rate	Convolution Range
MDCG1	3	1	1	3
MDCG2	5	2	1	5
MDCG 3	5	4	2	9
MDCG 4	9	8	2	17
MDCG 5	11	15	3	31
MDCG 6	15	28	4	57
MDCG 7	21	40	4	81
MDCG 8	23	55	5	111
Conv Layer 1	3	1	1	3
Conv Layer 2	1	0	1	1
Conv Layer 3	1	0	1	1
Conv Layer 4	3	1	1	3

**Table 2:** Differences between CGAN and ACGAN.

Model	Main Task	Auxiliary Task	Ultimate Goal
CGAN	Discriminating the classification	Discriminating the source	Getting discriminator *D*
ACGAN	Discriminating the source	Discriminating the classification	Getting generator *G*

**Table 3:** The CWRU dataset.

Fault Class	N	IR1	IR2	IR3	BF1	BF2	BF3	OR1	OR2	OR3
Number of training samples	448	376	372	374	373	375	375	375	374	376
Number of test samples	100	100	100	100	100	100	100	100	100	100
Defect size (inch)	-	0.007	0.014	0.021	0.007	0.014	0.021	0.007	0.014	0.021

**Table 4:** Designed tasks on the self-built dataset.

Task Name	S1	S2	S3	S4	S5
Number of training samples for each class	50	100	200	400	800
Number of test samples for each class	400	400	400	400	400

**Table 5:** Comparison of various methods on the CWRU dataset.

Models	Method	Number of Fault Classes	Testing Accuracy
ICDSVM	[35]	11	97.91%
MC-CNN	[21]	4	99.61%
FMCNN	[36]	10	98.8%
Unsupervised learning	[37]	10	99.66%
MA-MSCNN	[24]	12	99.86%
Proposed model	CGAN	10	100.0%

**Table 6:** The accuracy of Model A and Model B.

Trail	Trail 1	Trail 2	Trail 3	Trail 4	Trail 5	Average Accuracy
Model A	69.100%	62.175%	64.125%	68.650%	60.250%	64.860%
Model B	81.025%	83.575%	68.825%	79.950%	78.825%	78.440%

**Table 7:** Compared models.

Comparison Item/Model	Model1	Model2	Model3	Model4	Model5
MDC	✗	✓	✓	✓	✓
SE	✓	✗	✓	✓	✓
Residual structure	✓	✓	✗	✓	✓
Training strategy	CGAN	CGAN	CGAN	Supervised learning	GAN+ Fine-tuning

## Data Availability

The self-built dataset used in the study is available on reasonable request.
